# Hypersensitivity pneumonitis among wind musicians – an overlooked disease?

**DOI:** 10.1080/20018525.2017.1351268

**Published:** 2017-08-07

**Authors:** Janne Møller, Charlotte Hyldgaard, Sissel Brix Kronborg-White, Finn Rasmussen, Elisabeth Bendstrup

**Affiliations:** ^a^ Department of Respiratory Diseases and Allergy, Aarhus University Hospital, Aarhus, Denmark; ^b^ Department of Radiology, Aarhus University Hospital, Aarhus, Denmark

**Keywords:** Hypersensitivity pneumonitis, wind instruments, mycobacteria, molds

## Abstract

Hypersensitivity pneumonitis (HP) is a complex pulmonary disorder mediated by the immune system and caused by various inhaled antigens against which the subject has previously been sensitized. In about 50% of the cases, the antigen is not identified. Identification and removal of the eliciting antigen is important for the prognosis.

We report two cases of HP caused by molds and atypical mycobacteria isolated from wind instruments. We present the first case of HP caused by bassoon playing and another case of HP caused by molds in a trombone. HP caused by fungi and bacteria in wind instruments may be much more common than previously thought.

HP caused by fungi and bacteria in wind instruments is probably underdiagnosed; this calls for more clinical attention when HP is suspected.

## Introduction

Hypersensitivity pneumonitis (HP) is a complex pulmonary disorder mediated by the immune system and caused by various inhaled antigens against which the subject has previously been sensitized. HP is most often seen after inhalation of bird proteins, causing ‘Bird Fanciers Lung’, or fungal spores (*Saccharopolyspora rectivirgula* and *Thermoactinomycetes vulgaris*), causing ‘Farmer’s Lung’.[1] In about 50% of the cases, the antigen is not identified despite meticulous scrutiny of exposure history.[2] Identification of the eliciting antigen requires knowledge of occupational tasks and environment at home or at work but also at recreational activities. Identification and elimination of the eliciting antigen is highly important for the prognosis.[2]

We report two cases of HP; one caused by molds and one due to atypical mycobacteria isolated from wind instruments. Previous case reports have described a saxophone, a trombone and a bagpipe as the source of bacterial or fungal growth; one case had a fatal outcome. We present the first case of HP in a bassoon player and another case of HP caused by mold in a trombone. HP caused by fungi and bacteria in wind instruments may be much more common than previously thought. Presenting these two cases, we hope to attract clinical attention to the role of wind instruments in the development of HP.

## Case I

**Figure 1.**
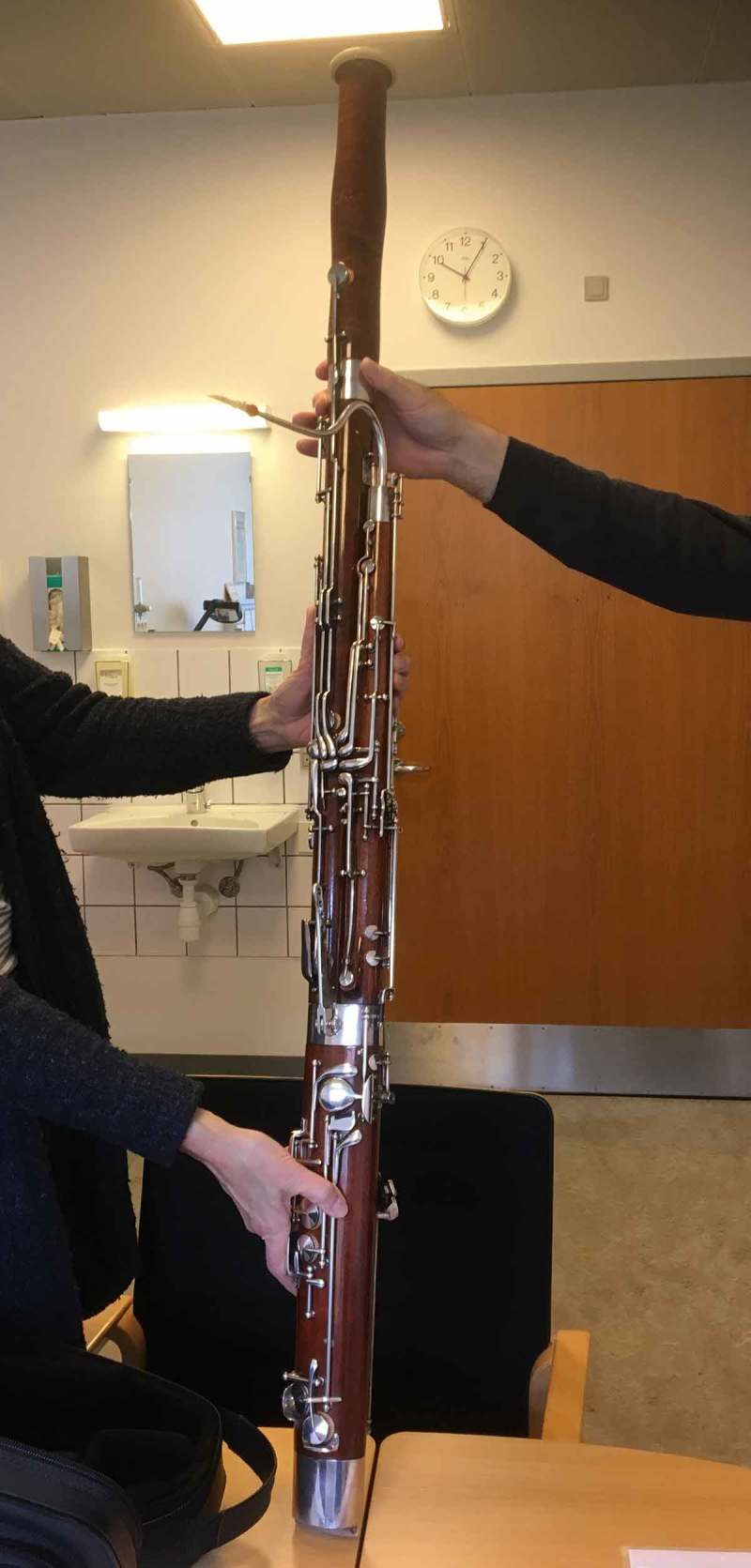
Basoon

A 56-year-old woman was referred to the Department of Respiratory Diseases and Allergy, Centre for Interstitial Lung Diseases at Aarhus University Hospital in 2016 due to cough and shortness of breath. She had never smoked. Onset of symptoms one year before had been ascribed to the disposal of a moldy carpet. The patient complained of persistent cough and dyspnea of varying intensity, although exposure to molds had stopped one year before and despite anti-asthmatic treatment initiated by her general practitioner. The patient was a professional bassoonist in a symphony orchestra (Figure 1). She reported that her respiratory symptoms had disappeared during a four-week summer holiday, where she had not been playing the bassoon.

**Figure 2.**
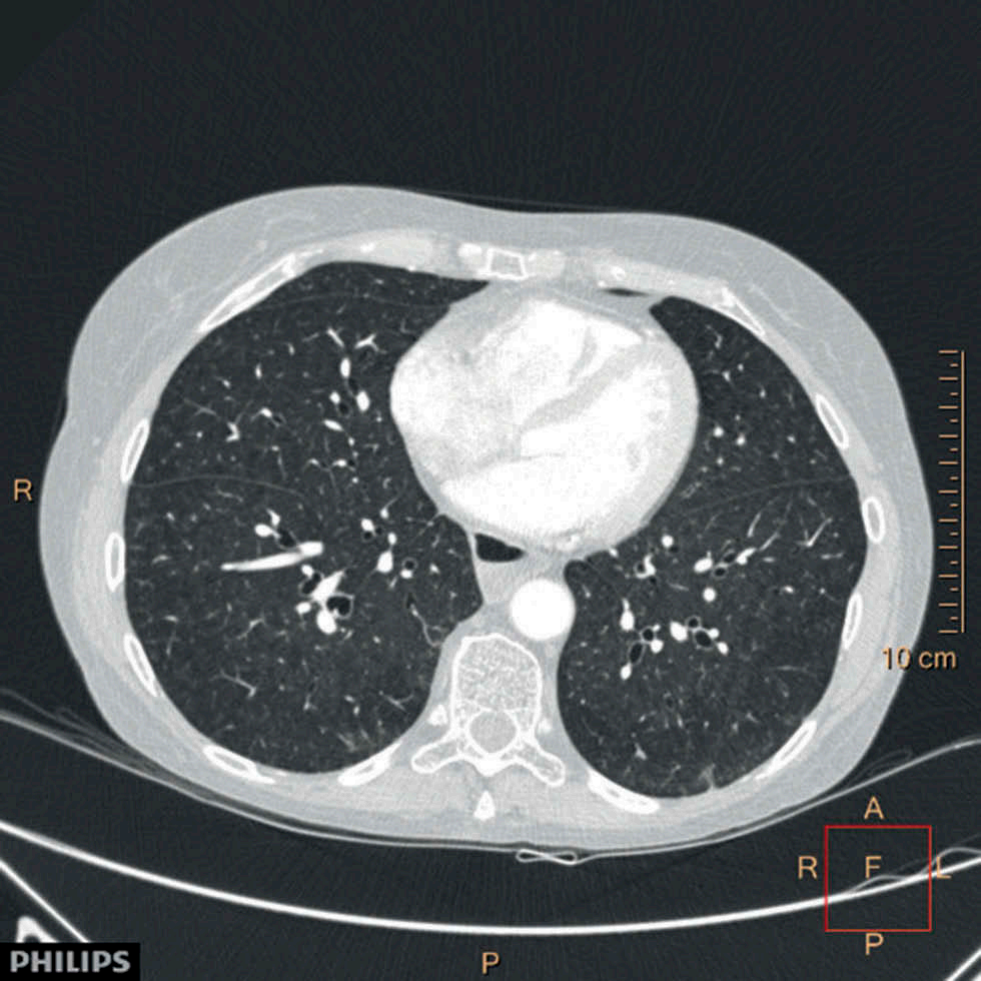
HRCT case I

Her physical examination revealed nothing abnormal. Pulmonary function test (PFT) demonstrated a mild obstruction with forced expiratory volume in one second (FEV1) of 2.21 l (71% predicted), forced vital capacity (FVC) of 3.99 l (111% predicted) and FEV1/FVC 55%. Total lung capacity was normal (101% predicted) and residual volume (RV) 125% predicted. Diffusing capacity for carbon monoxide (DLCO) was slightly reduced (76% predicted). High resolution computed tomography (HRCT) showed centrilobular nodules compatible with HP (Figure 2). Bronchoscopy with bronchoalveolar lavage (BAL) demonstrated a pronounced lymphocytic inflammation with 63% lymphocytes. No mycobacteria, bacteria, yeasts or molds were cultured.

*Mycobacterium chelonae, Phoma* and *Rhodoturola* were cultured from samples taken from the bassoon. The patient was diagnosed with HP due to exposure to atypical mycobacteria and fungi from her bassoon, typical HRCT features and lymphocytic alveolitis. She was advised to clean her instrument frequently. Steroid treatment was not indicated, as her PFT was almost normal.

## Case II

**Figure 3.**
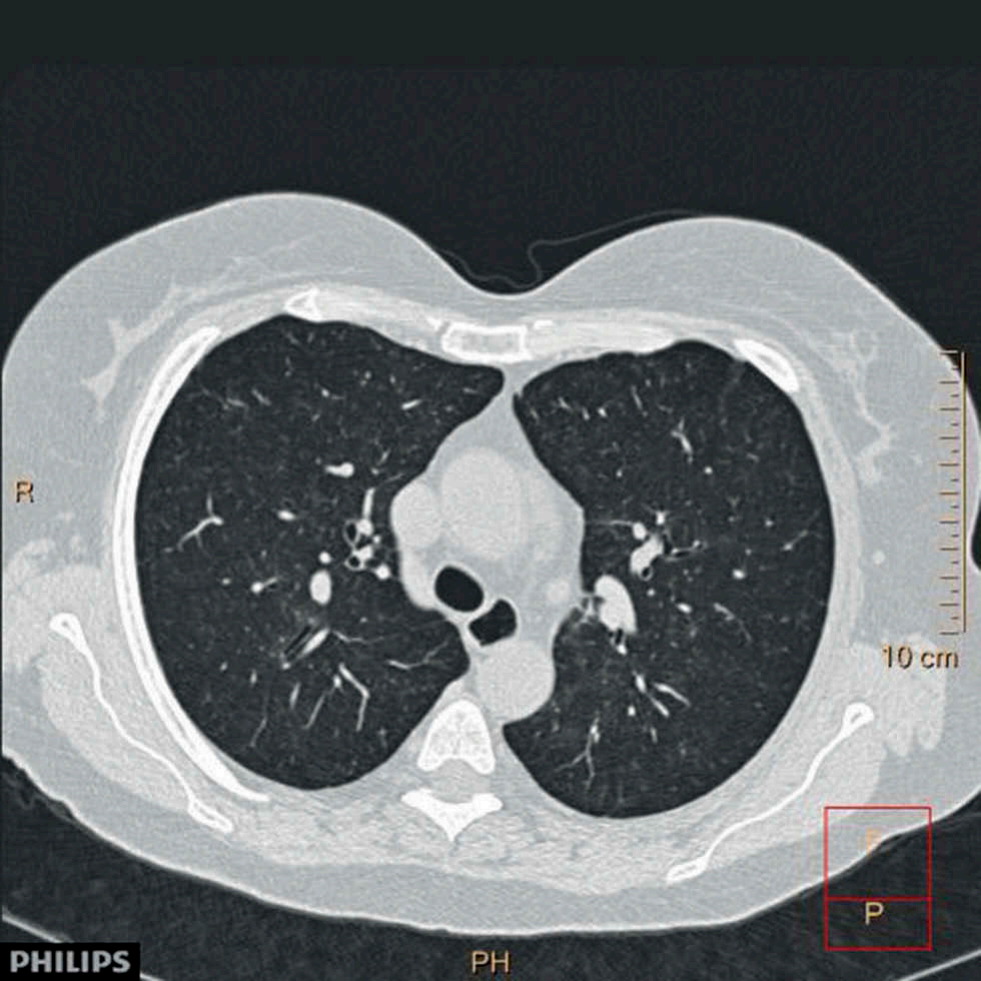
HRCT case II

A 64-year-old woman previously diagnosed with systemic sclerosis (SSc), Sjogren’s syndrome and bronchiectasis, was referred to the bronchiectasis clinic at the Department of Respiratory Diseases and Allergy at Aarhus University Hospital in 2014. The patient was not treated with any immunosuppressive drugs. PFT at referral showed FEV1 of 2.54 l (119% predicted), FVC of 3.39 l (133% predicted) and FEV1/FVC 75%. TLC was 108% predicted, RV 91% predicted and DLCO was reduced to 44% predicted. In 2014, an HRCT scan showed mild emphysema and sub-pleural reticulation. The patient was followed for 2 years, and after initiation of inhaled corticosteroids and long-acting beta-2-agonists she experienced a declining tendency to infections. In 2016 a chest CT was performed due to hemoptysis; it showed unchanged mild fibrosis but multiple centrilobular nodules and ground glass opacities (Figure 3). A bronchoscopy was macroscopically normal. Unfortunately, no BAL was performed. She was referred to the Department of Respiratory Diseases and Allergy, Centre for Interstitial Lung Diseases at Aarhus University Hospital on suspicion of SSc-related interstitial lung disease. She reported playing the trombone daily as a hobby, but found it increasingly difficult. PFT was stable with FEV1 2.60 l (127% predicted), FVC 3.41 l (140% predicted) and DLCO 45% predicted. Species of mold (*Paecilomyces ilacinus* and *Fusarium)* were cultured from samples taken from the trombone. Based on the history, exposure and HRCT, the patient was diagnosed with Trombone Player’s Lung, i.e. HP caused by molds from the trombone. She was advised to clean her instrument frequently to avoid recurrent exposure. At her next follow-up visit she was recovering from a long-term infection. PFT was generally unchanged.

## Discussion

We present the first case of HP caused by bassoon playing and the first case of Trombone Player’s Lung in a patient with scleroderma. HP is a rare disease with fewer than 50 annual cases in Denmark.[3] The pathogenesis of HP is not fully understood. Genetic susceptibility and host-related factors probably play a role, since only a minority of individuals exposed to potential antigens develop the disease. Smoking appears to have a protective effect.[1] The diagnosis is based on history of exposure, exposure-related symptoms, clinical presentation, HCRT and BAL findings, and histopathology if available.[4] The diagnosis of sub-acute HP is often uncomplicated, especially when the eliciting antigen is easily identified. Chronic HP can be more difficult to diagnose. PFT most often shows decreased diffusion capacity and a restrictive pattern, but a minority, including never smokers, experience obstruction,[5] as in case I. The exposure history in chronic HP may be subtle, and in many cases no eliciting antigen can be identified. HRCT findings may be less specific, with patterns of fibrosis indistinguishable from other interstitial lung diseases, and BAL does not always show lymphocytosis.[1] Elimination of the eliciting antigen is paramount for a more favorable disease course.[2] Systemic steroids are often used, and the acute and sub-acute forms of HP usually respond well. Steroid treatment is less beneficial in chronic HP, which emphasizes the importance of antigen identification and avoidance.

Previous cases have described HP caused by molds and atypical mycobacteria in wind instruments such as a trombone, a saxophone and a bagpipe. In the case of Trombone Player’s Lung, *Mycobacterium chelonae* and *Fusarium* species (molds) were identified in samples from the instrument.[6] Fungi such as *Candida albicans, Candida famata* and species of Cryptococcus were grown from a saxophone in Saxophone Player’s Lung.[7,8] Recently, *Paecilomyces variotti, Fusarium oxysporum, Penicillium species, Rhodotorula mucilaginosa, Trichosporon mucoides*, pink yeast and *Exophiala dermatitidis* were cultured from a bagpipe.[9]

The case reports are all examples of HP triggered by microbial growth in fluid and saliva found in wind instruments. In all cases, the respiratory symptoms disappeared when patients went on holiday not playing their instruments. Except one fatal case of Bagpipe Player’s Lung with continued exposure and no response to heavy immunosuppressive treatment, all patients improved after initiating thorough and regular cleaning of their instruments.

HP caused by molds in wind instruments is probably underdiagnosed. The exposure is often not work-related and may only be revealed if the patient is asked specifically. Professional musicians may be misdiagnosed with other disorders such as asthma and vocal cord papillomas. A study of seven wind musicians’ instruments revealed molds and mycobacteria associated with HP in all seven cases. Several of the musicians had respiratory symptoms.[6] Another study reported frequent colonization of positive cultures of potentially pathogenic molds in 13 of 15 saxophones examined, although none of the players had evidence of HP.[8] It has been suggested that the amount of air inhaled though the instrument affects the risk of developing HP. Some wind musicians mostly inhale the surrounding air, and only to a lesser degree the air that passes through the instrument. Thus, they are less exposed to contaminated air.[9] The finding of molds and mycobacteria in the present cases could be incidental. But the relief of symptoms during summer holidays in case I, difficulties when playing the trombone in case II combined with the HRCT findings in both cases and BAL lymphocytosis in case I make the diagnosis of HP highly likely. Improvement of the condition after cleaning the wind instruments will further support the diagnosis.

The present examples of mycobacterial and fungal growth in wind instruments causing HP lead us to believe that HP is more common among wind musicians than previously thought. This study, as well as the previous reports, suggests that any type of wind instrument can be contaminated with microbes associated with HP. This highlights the importance of a thorough history of environmental and occupational exposures as part of the diagnostic work-up when HP is suspected.

## Conclusion

HP caused by fungi and bacteria in wind instruments is probably underdiagnosed; this calls for more clinical attention when HP is suspected.
